# Identification of Potential Drug Targets for Immunoglobulin A Nephropathy: A Mendelian Randomization Study

**DOI:** 10.3390/biomedicines13030581

**Published:** 2025-02-25

**Authors:** Limei Xiong, Hui Zhang, Yannan Guo, Yuhong Tao

**Affiliations:** 1 Division of Nephrology, Department of Pediatrics, West China Second University Hospital, Sichuan University, Chengdu 610041, China; lmxiong1994@foxmail.com (L.X.);; 2 Key Laboratory of Birth Defects and Related Diseases of Women and Children (Sichuan University), Ministry of Education, Chengdu 610041, China

**Keywords:** druggable genes, DNA methylation, immunoglobulin A nephropathy, Mendelian randomization analysis, genetic susceptibility

## Abstract

**Background**: The current pharmacological treatments for Immunoglobulin A nephropathy (IgAN) demonstrate limited effectiveness and may cause serious side effects. This study aimed to explore novel potential drug targets for IgAN. **Methods**: We utilized summarized data from a recent genome-wide association study on IgAN, cis-expression quantitative trait loci data for druggable genes obtained from the eQTLGen Consortium, and DNA methylation quantitative trait loci data derived from the GoDMC database. Two-sample Mendelian randomization (MR) analysis, Bayesian colocalization, and mediation analysis through a two-step MR approach were performed to investigate their causal relationships. **Results**: Two-sample MR and colocalization analyses demonstrated that the expression of *HLA-DPA1* and *C4A* was associated with an increased risk of IgAN. In contrast, *TUBB*, *CYP21A2*, and *C4B* were associated with a decreased risk of IgAN. Mediation analysis revealed that the expression of *HLA-DPA1* acted as a mediator in the potential causal relationship between three DNA methylation sites (cg01140143, cg08898074, and cg12168509) and IgAN, with mediated proportions of 33.74% (95% CI 1.64–73.27), 41.67% (95% CI 20.78–66.97), and 50.34% (95% CI 27.89–74.76), respectively. **Conclusions**: Several druggable genes and DNA methylation sites were identified to show potential causal associations with IgAN risk and may be targeted for drug development. Nevertheless, additional experimental validation is warranted to clarify the specific roles of DNA methylation and the identified druggable genes in the pathogenesis of IgAN.

## 1. Introduction

Immunoglobulin A nephropathy (IgAN) represents the most prevalent form of primary glomerulonephritis. Unlike other common glomerular diseases such as membranous nephropathy, focal segmental glomerulosclerosis, and diabetic nephropathy, IgAN is characterized by the deposition of immune complexes, primarily immunoglobulin A (IgA), within the mesangial region [1]. The principal clinical manifestations include hematuria and proteinuria, accompanied by a progressive deterioration in renal function. It has been reported that IgAN in approximately 25–40% of the patients may progress to end-stage renal disease within 20–25 years and is a significant contributor to the incidence of end-stage renal disease [1,2,3]. The current KDIGO (Kidney Disease: Improving Global Outcomes) guidelines, published in 2021, underscore the importance of supportive care in the management of IgAN [4]. The measures include effective blood pressure control and the administration of angiotensin-converting enzyme inhibitors or angiotensin receptor blockers to mitigate proteinuria, which has been shown to have moderate efficacy. Furthermore, for patients classified as being at high risk for disease progression, the potential use of systemic glucocorticoids or immunosuppressive therapy should be considered.

While the conventional treatment methods referenced have demonstrated some efficacy in decelerating the progression of chronic kidney disease to end-stage renal disease, they have not succeeded in completely halting the progression of IgAN. In addition, these treatments may lead to serious side effects and toxicity. Therefore, halting or reversing the process of IgAN remains a challenge. With recent advancements in the comprehension of the pathophysiology of IgAN, several innovative targeted therapies have emerged, such as dual endothelin angiotensin receptor antagonists and targeted-mucosa-release budesonide [5,6,7,8]. This development signifies a transition in the management of IgAN from traditional immunosuppressive strategies to an era of targeted treatment. Consequently, the identification of new therapeutic targets that can prevent IgAN or delay its progression is of paramount significance.

IgAN is a complex autoimmune disease. The multiple-hit pathogenesis model of IgAN is widely acknowledged for its integration into genetic, biochemical, and clinical research [9]. Recent genome-wide association studies (GWAS) have established that susceptibility to this disease is significantly influenced by various common variants within the antigen processing and presentation pathway, thereby indicating a robust genetic foundation for IgAN [10,11,12]. Additionally, recent investigations have revealed that abnormal epigenetic modifications, particularly DNA methylation (DNAm), play a critical role in the pathogenesis and progression of autoimmune diseases such as systemic lupus erythematosus (SLE) and rheumatoid arthritis (RA) [13,14,15,16]. However, the precise pathogenic mechanisms underlying these associations and the specific situation in IgAN have yet to be fully elucidated.

Mendelian randomization (MR) is an innovative approach for causal inference by primarily utilizing single nucleotide polymorphisms (SNPs) identified through GWAS as instrumental variables (IVs) to evaluate the causal associations between exposures and outcomes. MR, in contrast to observational studies, could obtain more robust causal inferences by employing the principle of natural random allocation and mitigating the influence of confounders [17]. Compared to randomized controlled trials, MR analysis provides similar statistical power yet can effectively mitigate the effects of confounding variables and eliminate the risk of reverse causality [18,19]. This method can potentially lessen the need for expensive and iterative experiments, thereby breaking down the long-standing resource barrier in biomarker discovery. Recently, it has gained prominence in the area of target drug development and screening potential drug targets.

In this study, we aim to employ a two-sample MR analysis integrating summarized cis-expression quantitative trait loci (cis- eQTL) data for druggable genes and GWAS data for IgAN to assess the potential causal relationship between druggable genes and the risk for IgAN. Given that DNAm plays a critical role in influencing genomic signatures and modifying gene expression, we implemented a two-step MR analysis to explore the mediating pathway from DNAm to IgAN via the expression of druggable genes.

## 2. Materials and Methods

### 2.1. Study Design

**Figure 1:**
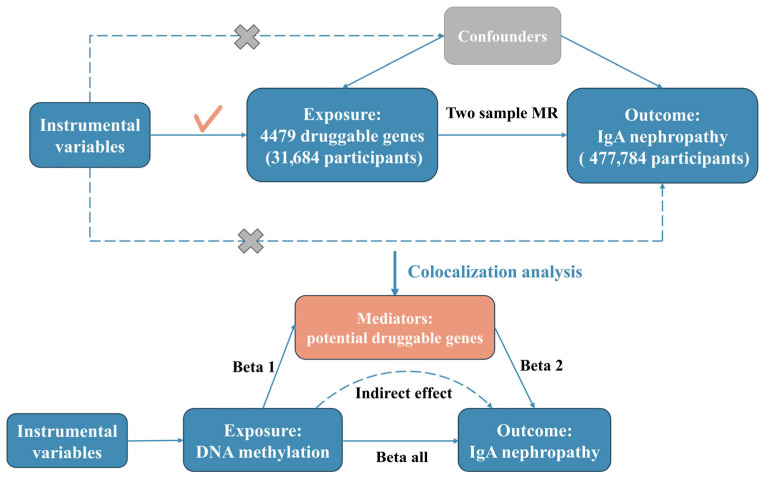
The flowchart illustrating the design of our study is presented herein. The abbreviations used are as follows: MR—Mendelian randomization; Beta 1—the total effect of DNA methylation on a potential druggable gene; Beta 2—the total effect of the potential druggable gene on IgA nephropathy; Beta all—the total effect of DNA methylation on IgA nephropathy which can be decomposed into: (i) indirect effect = Beta 1 × Beta 2, using a two-step approach and the product method, and (ii) direct effect = Beta all − Beta 1 × Beta 2. The proportion mediated is defined as the ratio of the indirect effect to Beta all.

This study comprised three primary components, as illustrated in Figure 1. First, a two-sample MR analysis was designed to explore the causal relationship between the expression levels of druggable genes and the susceptibility to IgAN. This analysis utilized SNPs derived cis-eQTL of the druggable genes in blood as IVs. Second, a colocalization analysis was performed to identify the presence of a common causal variant shared between the expression of druggable genes that exhibited significant MR results in the first step and the IgAN. Third, we performed a mediation analysis employing a two-step MR approach, which integrated summarized GWAS data for IgAN, DNAm, and the expression of potentially druggable genes. This analysis aimed to investigate the underlying epigenetic mechanisms of gene regulation in the bloodstream. For each identified druggable gene that showed strong colocalization significance with IgAN, we proceeded to the third step, which involved various MR tests. Initially, we assessed the causal relationships between genetically determined DNAm and the expression of potential druggable genes, utilizing SNPs derived from DNA methylation quantitative trait loci (DNA mQTL) in the blood as IVs, with DNAm serving as the exposure and cis-eQTL of druggable genes as the outcome. Subsequently, we analyzed the causal associations between genetically determined DNAm and IgAN, with DNAm as the exposure and IgAN as the outcome. Finally, we conducted a mediation analysis to identify potential mediators. In alignment with stringent inclusion and exclusion criteria, appropriate SNPs were identified as IVs. To guarantee the validity of the MR findings, all genetic variations employed as IVs must conform to three fundamental assumptions: (1) the selected IVs exhibit a direct association with the exposure; (2) the IVs must not exhibit correlation with potential confounding variables that could influence both the exposure and the outcome; and (3) the IVs affect the outcome exclusively through their effect on the exposure, rather than through alternative pathways.

### 2.2. Data Source

Finan et al. [20] identified a total of 4479 druggable genes, constituting the most comprehensive druggable genome library to date. Within this collection, 1427 genes are recognized as targets for approved pharmaceuticals and are currently in clinical development. Additionally, 682 genes encode proteins that are closely related to drug targets or are associated with drug-like compounds, while 2370 genes are categorized within significant druggable gene families or encode proteins that exhibit distant similarities to established drug targets. The cis-eQTL data about druggable genes in blood samples were sourced from the eQTLGen Consortium (https://www.eqtlgen.org/cis-eqtls.html, accessed on 15 August 2024). This consortium encompasses a total of 37 datasets and includes data from 31,684 individuals [21].

Genetic associations with IgAN were obtained from the largest and most recent publicly available GWAS [22]. The GWAS data for IgAN included 15,587 cases and 462,197 controls from the European population, with approximately 2.4 million SNPs analyzed. GWAS summary statistics for IgAN are publicly available from the GWAS Catalog, with the corresponding accession number being GCST90018866.

The DNA mQTL data can be accessed through the GoDMC database (http://mqtldb.godmc.org.uk/index, accessed on 15 August 2024), which encompasses 36 cohorts comprising a total of 27,750 European samples derived from whole blood [23]. In this study, a total of 420,509 DNA methylation sites were examined in conjunction with 10 million common genetic variants.

### 2.3. Selection of Instrumental Variables

To enhance the robustness and reliability of MR analysis, we implemented a series of rigorous quality control measures for the selection of IVs. First, we adopted a stringent criterion by applying the genome-wide significance threshold (*p* < 5 × 10^−8^) to identify SNPs associated with the exposure of interest. Second, to maintain the independence of our IVs, we utilized the clumping functionality of PLINK software (version 1.90) [24] and established a linkage disequilibrium threshold of r^2^ < 0.1, with a clumping distance of 10,000 kb. This clumping process was based on SNPs from druggable genes as defined by the 1000 Genomes Project European population [25]. Third, to confirm that the effects of SNPs on the exposure were consistent with the outcomes, we excluded any SNPs that were incompatible with the correlation between the exposures and outcomes. Additionally, we inferred positive strand alleles for palindromic SNPs based on allele frequencies or excluded palindromic SNPs directly when allele frequency data were unavailable. Finally, we calculated the F-statistic and excluded SNPs with F-statistics less than 10 to mitigate potential bias arising from weak IVs [26]. The F-statistics were computed via the following formula [27]:

$$F=\frac{R^{2}\times \left(N-k-1\right)}{k\times \left(1-R^{2}\right)}$$

*R*^2^ denotes the proportion of variance in exposure that can be accounted for by the chosen SNPs. In this context, *N* signifies the sample size, while *k* refers to the number of instrumental variables included in the analysis.

### 2.4. Statistical Analysis

#### 2.4.1. MR Analysis and Sensitivity Analysis

To assess the causal relationships between druggable genes and IgAN, we conducted a two-sample MR analysis. This MR analysis employed five distinct methodologies: inverse variance weighted (IVW) [28], weighted median [29], weighted mode, simple mode, and MR-Egger, utilizing the ‘TwoSampleMR’ package (version 0.5.11) [30]. The IVW method was designated as the primary analytical approach. To enhance the robustness of our analytical framework and to identify potential biases arising from ineffective IVs or horizontal pleiotropy, we incorporated MR-Egger, weighted median, simple mode, and weighted mode methods as supplementary analyses. The results of the MR analysis were presented as odds ratios (ORs) along with their corresponding 95% confidence intervals (CIs). We implemented false discovery rate (FDR) corrections to ascertain statistically significant MR outcomes, thereby addressing the challenges associated with multiple testing. A *p*-value (FDR corrected) < 0.05 was considered indicative of a significant causal relationship with outcome.

A sensitivity analysis was conducted to evaluate the potential presence of heterogeneity and horizontal pleiotropy within the data. Heterogeneity was considered present if Cochran’s Q-test *p*-value was less than 0.05 [31]; therefore, a random-effect IVW MR analysis should be used. We evaluated the possible existence of horizontal pleiotropy by employing MR-Egger regression, specifically analyzing its intercept term. A deviation from zero (*p* < 0.05) was interpreted as indicative of a directional pleiotropic bias [32]. Causality was regarded as significant when the following three criteria were satisfied: (1) the IVW *p*-value < 0.05, (2) there was no evidence of horizontal pleiotropy, and (3) the estimates derived from the IVW and supplementary Mendelian randomization analyses were consistent in their directional outcomes.

#### 2.4.2. Bayesian Colocalization Analysis

In the context of two-sample MR analysis, the associations between SNPs related to druggable genes and those associated with IgAN should be derived from a common SNP. To evaluate the reliability of the relationships between druggable genes and IgAN concerning a shared causal variant, a colocalization analysis was conducted employing a Bayesian framework, as outlined in reference [33]. This analysis was carried out using the R package “coloc” (version 5.2.3). The posterior probabilities (PPs) for five hypotheses (H0, H1, H2, H3, and H4) were computed to mitigate the risk of false-positive results. Notably, PPH4 indicates the presence of a shared causal variant that is associated with both the expression of druggable genes and IgAN. A PPH4 value exceeding 0.80 was interpreted as strong evidence supporting colocalization. Consequently, those druggable genes that exhibited significant causal effects on IgAN in the MR analysis and successfully passed the colocalization assessment are more likely to be considered viable drug targets for the treatment of IgAN.

#### 2.4.3. Mediation Analysis

A mediation analysis was conducted via two-step MR analysis, which aimed to determine whether the potentially druggable genes acted as mediating factors in the pathway linking DNAm to IgAN. The total effects of the identified gene-related DNAm on the IgAN encompassed two components: (1) the direct effects of the DNAm on IgAN, and (2) indirect effects mediated by the identified druggable genes, calculated through the product method (Beta 1 × Beta 2 in Figure 1) [34]. A 95% CI was determined by employing the delta method.

## 3. Results

### 3.1. Available Druggable Gene Data

To systematically identify potential drug targets for IgAN, 4479 human druggable genes identified by Finan et al. were considered [20]. However, upon querying the eQTLGen Consortium, only 2888 druggable genes had the cis-eQTL data from the database. Available druggable gene data are shown in Supplementary Table S1.

### 3.2. Mendelian Randomization Analysis Identified Potential Drug Targets for IgAN

**Figure 2:**
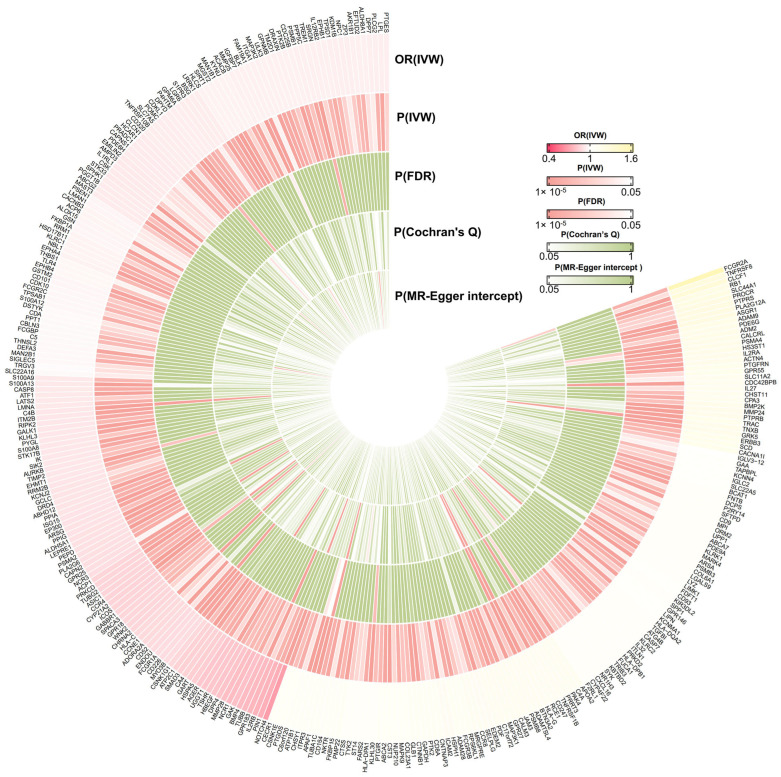
The cyclic heat map of the significant Mendelian randomization results of druggable genes on IgAN based on inverse variance weighted method with a *p*-value < 0.05. OR (IVW)—odds ratio based on inverse variance weighted method; P(IVW)—*p*-value based on inverse variance weighted method; P(FDR)—*p*-value of IVW method based on false discovery rate correction; P(Cochran’s Q)—*p*-value of Cochran’s Q test; P(MR-Egger intercept)—*p*-value of MR-Egger intercept.

Based on the IVW method, our results demonstrated that the expression levels of 310 druggable genes exhibited a significant association with susceptibility to IgAN with a *p*-value less than 0.05 (Figure 2). However, implementing FDR corrections, only 20 druggable genes exhibited a significant association with susceptibility to IgAN with *p*-value (FDR corrected) < 0.05. Although some of the results obtained from supplementary MR analyses were deemed insignificant based on the *p*-value < 0.05, their estimates exhibited a consistent direction in alignment with the IVW method. This consistency underscores the robustness of IVW.

**Figure 3:**
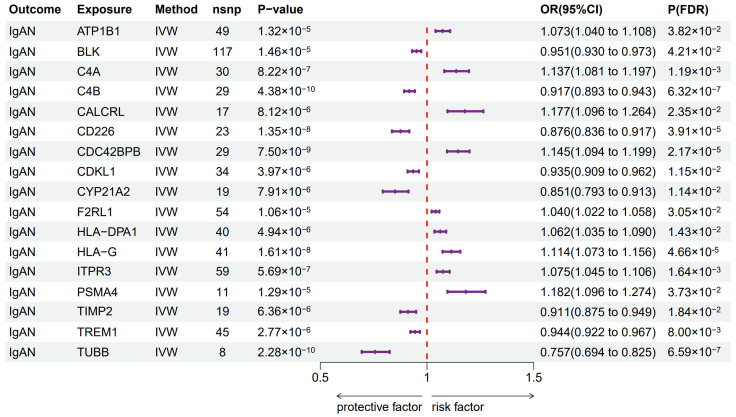
Significant Mendelian randomization results between the expression of druggable genes and IgAN susceptibility based on the IVW method after FDR correction. IVW—inverse variance weighted; P(FDR)—*p*-value of IVW method based on false discovery rate correction; nsnp—number of SNPs.

For three of these genes, the result of the MR-Egger intercept indicated the presence of horizontal pleiotropy with a *p*-value < 0.05, rendering their results in the IVW method unreliable. The outcomes of Cochran’s Q test indicated the presence of heterogeneity among the IVs for 10 genes. Finally, a total of 17 druggable genes showed a suggestive significant causal relationship with IgAN. Among these genes, the expression of nine genes (*F2RL1*, *HLA-DPA1, ATP1B1, ITPR3, HLA-G, C4A, CDC42BPB, CALCRL*, and *PSMA4*) was associated with an increased risk of IgAN, and the expression of the other eight genes (*TUBB, CYP21A2, CD226, TIMP2, C4B, CDKL1, TREM1* and *BLK*) was associated with a decreased risk of IgAN (Figure 3). The detailed results are shown in Table S2.

### 3.3. Colocalization Analysis

**Figure 4:**
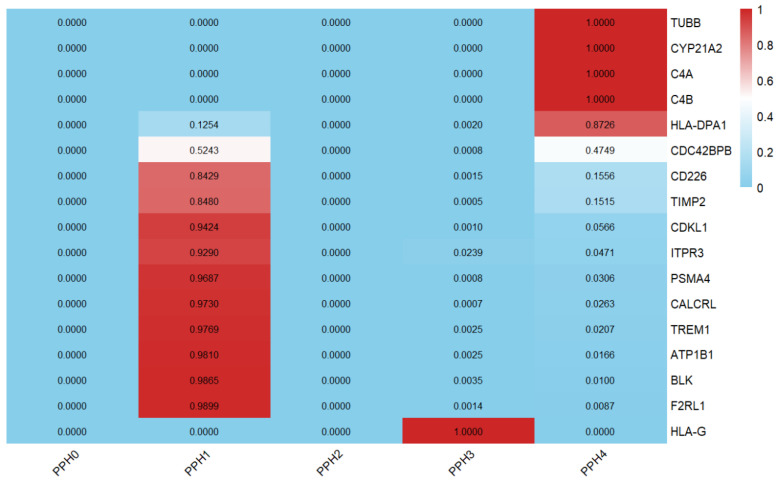
The results of colocalization analysis. PPH4 indicates the presence of a shared causal variant that is associated with both the expression of druggable genes and IgAN. A PPH4 > 0.80 was interpreted as strong evidence supporting colocalization.

Colocalization analysis was conducted to calculate the probability of shared causal variants between the expression of these 17 druggable genes exhibiting significant MR results and IgAN. The findings from the colocalization analysis revealed that five genes (*TUBB, HLA-DPA1, CYP21A2, C4A,* and *C4B*) were likely to share a causal variant with IgAN, as indicated by PPH4 greater than 80% (Figure 4).

### 3.4. Mediation Analysis

**Figure 5:**
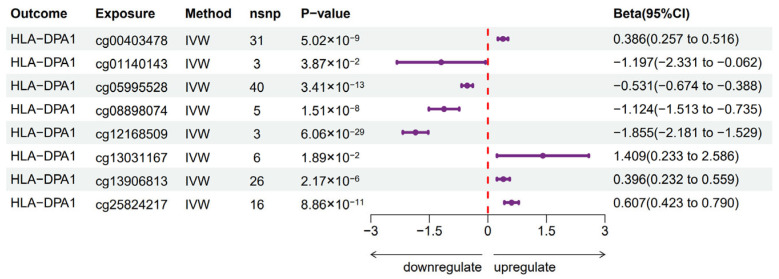
Significant Mendelian randomization results between *HLA-DPA1*-related DNA methylation and expression of *HLA-DPA1* based on IVW method. IVW—inverse variance weighted; nsnp—number of SNPs.

Methylation of DNA occurs at cytosine-phosphate-guanine (CpG) dinucleotides. DNAm data were generated using the Infinium HumanMethylation BeadChip (HumanMethylation450 or EPIC arrays) in the GoDMC database. Therefore, not all genes have corresponding CpGs in this database. Only *TUBB* and *HLA-DPA1* had related DNA mQTL data sourced from the GoDMC database. Using blood DNA mQTL and cis-eQTL data for *HLA-DPA1* and *TUBB*, we performed a two-sample MR analysis for each gene. The MR analysis revealed that eight CpG sites demonstrated significant causal effects on the expression of *HLA-DPA1*, as assessed by the IVW method, with a *p*-value < 0.05 (Figure 5). This finding was corroborated by consistent directional results from additional supplementary MR methodologies. The findings from the MR-Egger intercepts indicated a lack of substantial evidence for horizontal pleiotropy. The detailed results are shown in Table S3. In contrast, no significant causal effects were observed between CpGs and the expression of TUBB.

**Figure 6:**
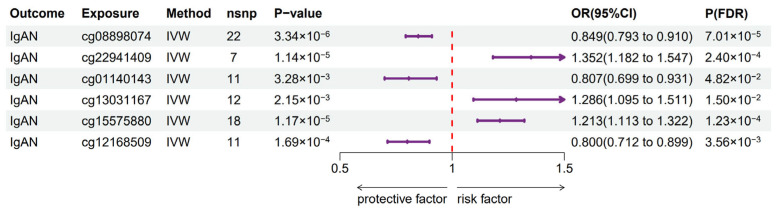
Significant Mendelian randomization results between *HLA-DPA1*-related DNA methylation and IgAN susceptibility based on the IVW method after FDR correction. IVW—inverse variance weighted; P(FDR)—*p*-value of IVW method based on false discovery rate correction; nsnp—number of SNPs.

Next, we used those DNA mQTL and GWAS summary data for IgAN to perform a two-sample MR analysis. MR analysis between *HLA-DPA1*-related DNAm data and IgAN revealed that 6 CpGs exhibited causal effects on IgAN, as determined by the IVW method, with *p*-value (FDR corrected) < 0.05 (Figure 6). This finding was also corroborated by consistent directional results from additional supplementary MR methodologies. The result of the MR-Egger intercept suggested no significant evidence of pleiotropy with all *p*-values  >  0.05. The detailed results are shown in Table S4. Therefore, this study demonstrated that both *HLA-DPA1*-related DNAm data and expression levels of *HLA-DPA1* exert causal effects on IgAN. Finally, we estimated the indirect effect of *HLA-DPA1*-related DNAm on IgAN via *HLA-DPA1* expression. Only three CpG sites (cg01140143, cg08898074, and cg12168509) exhibited the indirect effect on IgAN via the expression of *HLA-DPA1*, with the mediation effect of *HLA-DPA1* expression and mediated proportion shown in Table 1. As for TUBB-related DNAm data, there was no significant causal effect between CpGs and susceptibility to IgAN.

## 4. Discussion

In this study, we conducted multiple MR analyses to elucidate the intricate mechanisms associated with susceptibility to IgAN. Initially, we performed a comprehensive MR and colocalization analysis to assess the influence of the expression of 2888 druggable genes on IgAN susceptibility. This analysis identified the five most promising drug target genes, *TUBB, HLA-DPA1, CYP21A2, C4A,* and *C4B*, that may play a role in the pathogenesis of IgAN. Among them, *TUBB* and *HLA-DPA1* are novel druggable genes for IgAN, which have not been previously reported.

We further employed a two-step MR analysis to examine the relationship between DNAm and IgAN, specifically to determine whether this relationship is mediated by the aforementioned five potential druggable genes. Our findings indicated that the methylation levels of three *HLA-DPA1*-related CpG sites (cg01140143, cg08898074, and cg12168509) were associated with a reduced risk of IgAN. Furthermore, this effect was partially mediated through the expression of *HLA-DPA1*. Collectively, our findings propose a potential mechanism in which *HLA-DPA1* methylation leads to the downregulation of *HLA-DPA1* expression, which may contribute to a decreased risk of IgAN.

Our investigation revealed that the expression of the *HLA-DPA1* gene had a significant causal effect on susceptibility to IgAN. *HLA-DPA1* is classified within the HLA class II alpha chain paralogues. This class II molecule is expressed in antigen-presenting cells, including B lymphocytes, dendritic cells, and macrophages, and it is crucial for the immune response as it presents peptides that are derived from extracellular proteins. The HLA complex, also known as the major histocompatibility complex, has been generally associated with native kidney diseases for over five decades. Advances in technology and understanding of HLA biology and typing, along with improved case ascertainment, have led to a well-established recognition of the genetic associations between HLA and renal diseases [35]. In conjunction with various genetic and environmental factors, HLA allomorphs have emerged as significant risk factors in numerous immune-mediated renal disorders, including IgAN and membranous nephropathy [10,35,36]. Prior research has indicated that *HLA-DPA1* is associated with RA and SLE, and it may also contribute to the pathogenesis of diabetic kidney disease [37,38,39,40]. Our discovery in this study expands the previous findings and suggests that *HLA-DPA1* may be a contributing factor in the development of IgAN. Based on the functions of *HLA-DPA1*, we suppose that the *HLA-DPA1* may influence susceptibility to IgAN by regulating antigen presentation, enhancing the presentation of mucosal pathogen antigens, and activating autoimmune responses. The core mechanism may lie in immune dysregulation caused by abnormal antigen recognition, ultimately promoting immune complex deposition and kidney injury. In the future, further research is needed to explore the specific mechanisms of *HLA-DPA1* influencing IgAN susceptibility.

DNAm represents a prominent form of epigenetic modification that can influence genomic signatures and subsequently modify gene expression. Increasing evidence indicates that the dysregulation of DNAm is significantly involved in the pathogenesis of various human diseases, including monogenic epigenetic disorders, autoimmune conditions, metabolic disorders, hematologic malignancies, and solid tumors [13]. Prior research has demonstrated that alterations in DNAm contribute to the pathogenesis of RA and SLE by affecting immune cell function, which leads to increased inflammation and autoimmunity through the upregulation of specific genes [14,15,16]. In our investigation, MR and mediation analyses revealed that the DNAm of cg01140143, cg08898074, and cg12168509 in *HLA-DPA1* was associated with a reduced risk of IgAN susceptibility, with the causal effect being partially mediated by the downregulation of *HLA-DPA1* expression. Our findings provide novel insights into the potential role of DNAm in the involvement of *HLA-DPA1* in IgAN and suggest potential avenues for future therapeutic development targeting this condition. However, further research is required to elucidate the precise roles of DNAm and HLA genes in the pathogenesis of IgAN.

TUBB is a protein-coding gene that plays a crucial role in the formation of a heterodimer with α-tubulin, serving as a structural component of microtubules [41]. A recent study that employed multi-omics approaches to analyze the expression levels of TUBB found a positive correlation between TUBB expression and the abundance of CD4+ T helper 1 cells, which are characterized by the secretion of the proinflammatory cytokine interferon-gamma (IFN-γ) [42]. Additionally, the study indicated a negative correlation between TUBB mRNA expression levels and the abundance of B cells and CD8+ T cells. While these findings were primarily focused on cancer, the significant role of TUBB in immune modulation warrants attention. Nevertheless, there is a paucity of research examining TUBB in the context of kidney diseases. To date, only one in vitro study has demonstrated that the upregulation of TUBB expression can exacerbate inflammation, promote apoptosis, and inhibit autophagy in cases of acute kidney injury [43]. Therefore, we hypothesize that the TUBB gene may influence susceptibility to IgAN by influencing immune cell function and inflammatory signaling pathways. Further investigations are necessary to clarify the mechanisms by which TUBB influences susceptibility to IgAN.

In the context of MR analysis and colocalization analysis, three genes, *C4A, C4B,* and *CYP21A2*, exhibited a significant causal association with susceptibility to IgAN, which is consistent with the previous study [44]. *C4A* and *C4B* belong to the complement component C4 family. *C4A* encodes the acidic variant of complement factor 4, whereas C4B encodes the basic variant of the same factor; both are integral to the classical activation pathway. The activation of the complement system and the subsequent local inflammatory response are critical components in the pathogenesis of IgAN. Park et al. investigated gene expression alterations in the glomeruli of individuals with IgAN through RNA sequencing and reported a notable upregulation of *C4A* and *C4B* expression in the glomeruli of affected patients [45]. Therefore, we hypothesize that *C4A* and *C4B* genes may influence the occurrence and development of IgA nephropathy by regulating complement activation and immune complex metabolism. However, the precise functions of these two genes and the mechanisms contributing to the pathogenesis of IgAN require further investigation. *CYP21A2* encodes a member of the cytochrome P450 superfamily of enzymes. These cytochrome P450 proteins function as monooxygenases, facilitating numerous biochemical reactions that are critical for drug metabolism as well as the synthesis of lipids. The cytochrome P450 epoxygenase/epoxide hydrolase axis plays an important role in inflammation, vascular function, and metabolic regulation by regulating the production and metabolism of epoxyeicosatrienoic acids. A previous investigation assessed the plasma protein levels in patients with IgAN in comparison to healthy controls, utilizing whole blood cells and renal tissue [46]. The findings revealed that five cytochrome P450 proteins exhibited significantly elevated levels in both whole blood cells and kidney tissue of IgAN patients relative to the healthy control group. Therefore, the *CYP21A2* gene might play a role in the pathogenesis of IgAN through the cytochrome P450 epoxygenase/epoxide hydrolase axis, which might be a new intervention strategy for IgAN patients. However, additional research is warranted to elucidate the specific mechanisms underlying this association. Alongside *HLA-DPA1, TUBB*, *CYP21A2*, *C4A*, and *C4B*, our research identified an additional twelve targets that were not corroborated by colocalization analysis. Nonetheless, the potential significance of these targets should not be entirely missed, and they may offer extensive opportunities for the advancement of therapeutic agents for IgAN.

Our research presents several notable advantages. Our study pioneers the application of drug target MR analysis by utilizing cis-eQTL data and mitigating the risk of horizontal pleiotropy in the context of IgAN. While several MR analyses have been conducted to investigate potential drug treatment targets for IgAN, the majority of these studies have predominantly relied on summary data-based MR analyses [44,47,48]. Notably, only one prior study has conducted a proteome-MR analysis to examine the causal relationship between plasma proteins and IgAN and to identify several potential drug targets for this condition [48]. As far as we know, the GWAS data for IgAN and the eQTL data for druggable genes employed in our analysis represent the largest dataset currently available. To reduce the potential for bias and ensure the reliability of our MR findings, we implemented stringent screening criteria and confirmed adherence to essential assumptions. Through a two-sample MR analysis and colocalization analysis, we identified two novel druggable genes, *HLP-DPA1* and *TUBB*, which have not been previously reported in relation to IgAN. These genes were identified as potential therapeutic targets that warrant prioritization in further experimental or clinical investigation. In addition to identifying potential drug targets for IgAN, our study delves into the underlying epigenetic mechanisms, thereby making a substantial contribution to the understanding of DNA methylation regulation in IgAN. In summary, the significance of this study lies in bridging observational epidemiology with mechanistic research, identifying gene variations or loci strongly associated with IgAN, and ultimately accelerating the discovery of potential therapeutic targets for IgAN.

It is important to acknowledge several limitations inherent in our study. First, the analysis was constrained by the limited availability of druggable genes associated with cis-eQTL data for MR analysis. Thus, we might have missed some important genes that were not tagged in the cis-eQTL data. Besides, the application of FDR correction may have resulted in the exclusion of significant genes that were filtered out owing to FDR thresholds. Second, our MR analysis was exclusively based on eQTL data derived from blood samples, as eQTL data from renal tissues were not accessible. While blood can provide insights into the systemic interactions of biological processes, it may not fully capture the molecular signals pertinent to the disease, which could be more pronounced in other tissues. Third, although MR analysis integrates GWAS data related to IgAN, eQTL of druggable genes, and DNA mQTL, it can elucidate causal relationships only to a certain extent. However, the complexity of the actual biological context is further complicated by the influence of various factors, particularly environmental and non-genetic variables. For example, fine particulate matter < 2.5 μm and the deleterious compounds in tobacco smoke, including polycyclic aromatic hydrocarbons and heavy metals such as cadmium, have the potential to induce oxidative stress, which subsequently impacts the functionality of DNA methyltransferases. The oxidative stress can result in DNA damage and modify the availability of methyl donors, leading to either global hypomethylation or hypermethylation of specific genes. Consequently, it is important to examine the impact of the interaction between environmental factors and genetics on IgAN, which was not addressed in this study. Therefore, MR analysis should not be regarded as a substitute for clinical trials. Moreover, the identified drug targets and their interactions require additional experimental validation and clinical investigation to determine their clinical significance and therapeutic viability. Finally, it is important to note that our analysis was conducted using data from the European population, which may limit the generalizability of our findings to other ethnic groups.

## 5. Conclusions

This study, using MR and colocalization analyses, identified that the expression of *HLA-DPA1*, *TUBB*, *CYP21A2*, *C4A,* and *C4B* had a causal effect on susceptibility to IgAN. These genes may serve as potential targets for drug development. Our findings further elucidate a causal relationship between three *HLA-DPA1*-related DNAm sites and IgAN, with a portion of this effect being mediated by the expression of *HLA-DPA1*. However, further investigations are necessary to validate the causal relationship between these potential targets and IgAN and clarify the precise mechanisms by which the identified genetic variants/loci contribute to the etiology of IgAN.

## Figures and Tables

**Table 1:** The mediation effect of *HLA-DPA1*-related DNA methylation on IgAN via expression of *HLA-DPA1.*

CpG	Beta1 (95% CI)	Beta2 (95% CI)	Beta All (95% CI)	Indirect Effect (95% CI)	Proportion Mediated (95% CI)
cg01140143	−1.197 (−2.331, −0.062)	0.061 (0.035, 0.087)	−0.215 (−0.358, −0.072)	−0.072 (−0.157, −0.004)	33.74% (1.64, 73.27)
cg08898074	−1.124 (−1.513, −0.735)	0.061 (0.035, 0.087)	−0.163 (−0.232, −0.094)	−0.068 (−0.109, −0.034)	41.67% (20.78, 66.97)
cg12168509	−1.855 (−2.181, −1.529)	0.061 (0.035, 0.087)	−0.223 (−0.339, −0.107)	−0.112 (−0.167, −0.062)	50.34% (27.89, 74.76)

Beta1: the total effect of identified CpG on the expression of *HLA-DPA1*; Beta 2: the total effect of the expression of *HLA-PDA1* on IgA risk; Beta all: the total effect of identified CpG on IgA risk; Indirect effect = Beta1 × Beta2; Proportion mediated was the indirect effect divided by the total effect.

## Data Availability

The data presented in this study are openly available in the GWAS Catalog (https://www.ebi.ac.uk/gwas/), eQTLGen Consortium (https://www.eqtlgen.org/cis-eqtls.html) and GoDMC database (http://mqtldb.godmc.org.uk/index) (accessed on 15 August 2024).

## Supplementary Materials

The following supporting information can be downloaded at: https://www.mdpi.com/article/10.3390/biomedicines13030581/s1, Table S1: Available druggable genes and their locations; Table S2: Significant MR analysis results of causal links between 17 druggable genes and IgAN; Table S3: Significant MR analysis results between *HLA-DPA1*-related DNA methylation and expression of *HLA-DPA1*; Table S4: Significant MR analysis results between *HLA-DPA1*-related DNA methylation and IgAN.

## Abbreviations

The following abbreviations are used in this manuscript:

CI	Confidence interval
CpG	Cytosine-phosphate-guanine
DNAm	DNA methylation
DNA mQTL	DNA methylation quantitative trait loci
eQTL	Expression quantitative trait loci
FDR	False discovery rate
GWAS	Genome-wide association study
IgAN	Immunoglobulin A nephropathy
IV	Instrumental variable
IVW	Inverse variance weighted
MR	Mendelian randomization
OR	Odds ratio
PPs	Posterior probabilities
SLE	Systemic lupus erythematosus
SNPs	Single nucleotide polymorphisms
RA	Rheumatoid arthritis
